# Impact of an integrated care program on glycemic control and cardiovascular risk factors in patients with type 2 diabetes in Saudi Arabia: an interventional parallel-group controlled study

**DOI:** 10.1186/s12875-017-0677-2

**Published:** 2018-01-02

**Authors:** Ayla M. Tourkmani, Osama Abdelhay, Hesham I. Alkhashan, Aboud F. Alaboud, Ahmed Bakhit, Tarek Elsaid, Ahmed Alawad, Aljohara Alobaikan, Hala Alqahtani, Abdulaziz Alqahtani, Adel Mishriky, Abdulaziz bin Rsheed, Turki J. Alharbi

**Affiliations:** Family and Community Medicine Department, Prince Sultan Military Medical City, P.O. Box 7897, Riyadh, 11159 Saudi Arabia

**Keywords:** Type 2 diabetes, Cardiovascular risk, Glycemic control, Multidisciplinary care

## Abstract

**Background:**

Long intervals between patient visits and limited time with patients can result in clinical inertia and suboptimal achievement of treatment goals. These obstacles can be improved with a multidisciplinary care program. The present study aimed to assess the impact of such a program on glycemic control and cardiovascular risk factors.

**Methods:**

In a randomized, parallel-group trial, we assigned 263 patients with poorly controlled type 2 diabetes mellitus (T2DM) to either a control group, standard care program, or a multidisciplinary care program involving a senior family physician, clinical pharmacy specialist, dietician, diabetic educator, health educator, and social worker. The participants were followed for a median of 10 months, between September 2013 and September 2014. Glycated hemoglobin (HbA1c), fasting blood glucose (FBG), lipid profiles, and blood pressure (BP) were measured. The assignment was blinded for the assessors of the study outcomes. The study registry number is.

**Results:**

In the intervention group, there were statistically significant (*p* < 0.05) post-intervention (relative) reductions in the levels of HbA1c (−27.1%, 95% CI = −28.9%, −25.3%), FBG (−17.10%, 95% CI = −23.3%, −10.9%), total cholesterol (−9.93%, 95% CI = −12.7%, −7.9%), LDL cholesterol (−11.4%, 95% CI = −19.4%, −3.5%), systolic BP (−1.5%, 95% CI = −2.9%, −0.03%), and diastolic BP (−3.4%, 95% CI = −5.2%, −1.7%). There was a significant decrease in the number of patients with a HbA1c ≥10 (86 mmol/mol) from 167 patients at enrollment to 11 patients after intervention (*p* < 0.001). However, the intervention group experienced a statistically significant increase in body weight (3.7%, 95% CI = 2.9%, 4.5%). In the control group, no statistically significant changes were noticed in different outcomes with the exception of total cholesterol (−4.10%, *p* = 0.07). In the linear regression model, the intervention and the total number of clinic visits predicted HbA1c improvement.

**Conclusions:**

Implementation of a patient-specific integrated care program involving a multidisciplinary team approach, frequent clinic visits, and intensified insulin treatment was associated with marked improvement in glycemic control and cardiovascular risk factors of poorly controlled T2DM patients in a safe and reproducible manner.

**Trial registration:**

ISRCTN Identifier: ISRCTN83437562 September 19, 2016 Retrospectively registered.

**Electronic supplementary material:**

The online version of this article (10.1186/s12875-017-0677-2) contains supplementary material, which is available to authorized users.

## Background

Saudi Arabia has one of the highest rates of diabetes in the world [1]_._ Local population studies estimate the prevalence of diabetes at approximately 24% among Saudi adults [2]. This is approximately three times the world average [1]. A recent epidemiologic forecast study that incorporated the high obesity and smoking prevalence trends among Saudi adults estimated type 2 diabetes mellitus (T2DM) at 44% in 2022 [3]. In addition to the associated increased risk of morbidity and mortality, T2DM among Saudis has led to a surge in healthcare utilization and allocated costs [4]. Diabetes is known to increase the risk of vascular diseases such as heart diseases and stroke markedly [5]. This can be averted, or at least delayed, by intensive glycemic control [6, 7], along with the control of associated risk factors such as hypertension and dyslipidemia [7–9]. However, the compliance with these preventive measures by patients with T2DM is inadequate [10, 11].

Primary care physicians manage most patients with T2DM. However, long intervals between patient visits and limited time with patients can result in clinical inertia and, consequently, suboptimal achievement of treatment goals [12, 13]. Several strategies have been described to overcome barriers to efficient diabetes management at primary care settings, including a multidisciplinary team approach [12, 14]. The implementation of such an approach was successful in improving diabetes care in primary care patients [15]. We have reported a successful integrated care program for improving diabetes management in Saudi Arabia [16]. However, the small sample size and the lack of control limited the inferences from the study findings. The aim of the current study was to evaluate the impact of a multidisciplinary diabetic care program on glycated hemoglobin (HbA1c) and cardiovascular risk factors among patients with poorly controlled T2DM in a primary care setting, using a controlled interventional design. The study assessed changes in HbA1c, fasting blood glucose (FBG), total cholesterol, triglycerides, HDL cholesterol, LDL cholesterol, systolic and diastolic blood pressure (BP), body weight, number of visits, and record of concomitant medications and the frequency of adjustment.

## Methods

### Setting

The study was conducted in Al-Wazarat Chronic Diseases Center, a division of the Al-Wazarat Health Care (WHC) Family Medicine Center in Riyadh, Saudi Arabia. The Chronic Diseases Center consists of 12 specialized clinics, primarily for patients with T2DM, hypertension, dyslipidemia, and bronchial asthma, in addition to a procedures room and support services such as pharmacy, laboratory, and radiology. The Chronic Diseases Center is staffed by senior family physicians who are board certified and/or specialized in diabetes care, a board certified clinical pharmacist, dieticians, diabetic educators, health educators, and social workers. The daily clinics are run by six physicians serving approximately 120 patients daily.

### Design

A randomized, controlled interventional study was conducted between September 2013 and September 2014. Enrollment data were assessed by reviewing the patient charts for at least two visits before joining the study. Outcome data were assessed by prospectively following patients for at least two visits after joining the study (for a maximum of 9 months). Controls were recruited from the same center using the same eligibility criteria. All required ethical approvals from the local ethical committee were obtained before data collection.

### Population and eligibility

**Fig. 1:**
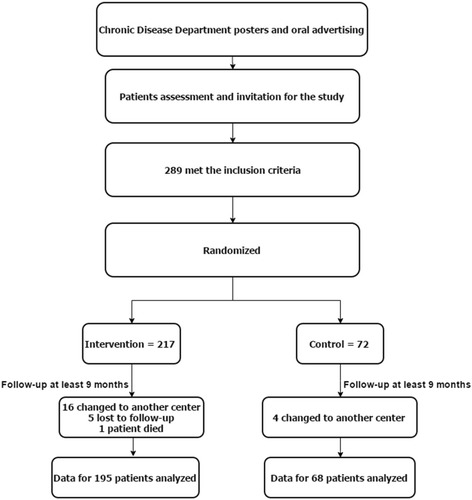
Flow diagram of the progress in a randomized controlled trial

The study sample was recruited from adult patients, 18 years or older, with T2DM who received their diabetic care at the Chronic Diseases Center of WHC. Patients who had at least two clinic visits before joining the study and were able to provide informed consent were checked for eligibility for enrollment. Patients who received care from both diabetes clinics at the endocrinology department and primary care clinics were excluded to avoid double care and to assure a standardized level of management. The eligibility criteria included one or more of the following: (1) poor glycemic control (HbA1c >10 [86 mmol/mol] or persistent elevation of HbA1c >8 [64 mmol/mol] for 1 year or more); (2) failure to respond to therapeutic insulin dose of >2 units/kg or 200 units irrespective of weight; (3) inadequate adherence to insulin; (4) uncontrolled hypertension or hyperlipidemia with maximum possible combination of medications; (5) comorbidity such as cardiovascular, renal, or hepatic disease; and (6) inadequate continuity of care (such as recurrent missed appointments for insulin titration). The eligible patients were consecutively assigned to either the intervention or control groups using a computerized random number generator. The 289 patients were assigned unique study numbers ranging from 1 to 289. The number assigned was consistent with the recruitment date (i.e., the first patient recruited was assigned the number 1 and the last patient recruited was assigned the number 289). The clinical pharmacist who acted as the case manager conducted assigned study numbers. The biostatistician generated a random sequence of 72 numbers out of 289 using a computer program without knowing the order of the patients. The case manager assigned the patients’ numbers who matched those on the random sequence to the control group. The recruitment and randomization processes of the patients are illustrated in Fig. 1.

### Sample size

Considering the results of the pilot study, we proposed that an integrated care program can reduce HbA1c by 3 points and FBG by 3 mmol/L. To detect a 2-point difference in HbA1c (3.0 versus 1.0 with a standard deviation [SD] of 2.7) between the intervention and control groups with 80% power and 95% confidence, 80 patients were required (60 in the intervention and 20 in the control group, assuming a ratio of 3:1). Similarly, to detect a difference of 2 mmol/L in FBG (3.0 versus 1.0 with a SD of 5.0) between the intervention and control groups with 80% power and 95% confidence, 264 patients were required (198 in the intervention and 66 in the control group, assuming a ratio of 3:1). Therefore, the larger sample size was adopted. The researchers opted to select a smaller size for the control group compared with the intervention (1 to 3) to maximize the number of patients gaining potential benefits from the intervention.

### Outcome

The absolute and relative changes in the levels of HbA1c, FBG, blood lipids (total, LDL, HDL cholesterol, and triglycerides), BP (systolic and diastolic), and body weight during the study relative to baseline were the outcomes measured.

### Intervention

The integrated care program is a multidisciplinary program used for the care of patients in the intervention group. Patients were referred from any discipline working in the Chronic Diseases Center when they fulfilled the eligibility criteria for the integrated care program to the case manager. The program team included a senior family physician, clinical pharmacy specialist who acted as a case manager, dietician, diabetic educator, health educator, and social worker. The program team met once or twice weekly to review the eligibility of referred patients and to assess and decide on the care plans for those who had already been enrolled. The care provided was the standard care per the guidelines of the American Diabetes Association (ADA) [17], but intensified with consideration for individual clinical and social factors. The case manager was responsible for arranging required appointments with other specialties as per the care plan, as well as evaluating the compliance and adverse effects of the new plan, through at least weekly appointments in the first 3 months. Enrolled patients had to be seen at least once by all members of the program team during the period of enrollment, with the exception of the social worker who was seen on an as-needed basis. Strategies to improve the care were patient-based and included (but not limited to) providing more clinic visits, frequent monitoring of outcomes, improving multidisciplinary communication and coordination, providing additional diabetic education and dietetic advice, promoting self-management, providing a booklet for home blood glucose monitoring, adjusting doses according hepatic and renal functions, assessing the need and performing insulin titration, encouraging medication adherence, providing social support, sending patients reminders, and making telephone calls [12, 14, 18].

### Standard care

**Fig. 2:**
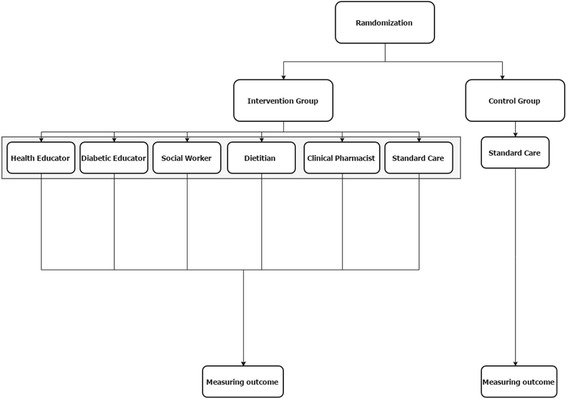
Graphical depiction of the intervention

The care provided to the patients in the control group was congruent with the ADA guidelines [17] with regular appointments every 3 months in the Chronic Disease Clinics. (Additional file 1: Table S1). The intervention is illustrated in Fig. 2.

### Statistical methods

Patients’ characteristics are described as means and SDs for continuous data and frequencies and percentages for categorical data. Significant differences between the intervention and control groups were tested using a Student’s *t*-test or Mann–Whitney test (as appropriate) for continuous data and chi-square test or Fisher’s exact test (as appropriate) for categorical data. The percentage of change in the study outcomes was defined as the amount of change during the study relative to baseline at enrollment. The change in the levels of the study outcomes was examined using a paired *t*-test. The correlations between the change in HbA1c and the patient’s age and clinical and management factors were examined using Spearman’s correlation. Independent predictors of HbA1c change were evaluated using a multivariate linear regression model. All *p*-values were two-tailed. *P*-values <0.05 were considered significant. SPSS software (release 20.0, SPSS Inc., Chicago, U.S.) was used for all statistical analyses.

## Results

The final study analysis included 263 patients with T2DM, with 195 patients in the intervention group and 68 patients in the control group. The demographic and clinical characteristics of both groups at the study enrollment are described in Table 1. The mean age was roughly similar in both groups (56.9 ± 12.0 years in the intervention group versus 57.7 ± 11.6 years in the control group). Females similarly represented the majority of patients in both groups (65.6% versus 63.2%). The intervention group had a significantly lower number of comorbidities compared with the control group (2.3 ± 0.8 versus 3.0 ± 1.0, *p* < 0.001), with lower rates of hypertension (30.3% versus 70.6%, *p* < 0.001) and dyslipidemia (46.2% versus 95.6%, *p* < 0.001). However, the intervention group had significantly higher body weights (82.9 ± 17.6 versus 74.8 ± 14.3, *p* < 0.001) and HbA1c (11.2 ± 1.4 [99 mmol/mol (84–114)] versus 10.1 ± 1.6 (87 mmol/mol [69–104], *p* < 0.001) compared with the control group. Both groups had similar levels of fasting blood glucose, blood lipids, and blood pressure.

**Table 1:** Demographic and clinical data of the study groups at enrollment

	Intervention (*N* = 195)	Control (*N* = 68)	*p*-value^a^
Age (years)			
< 50	47 (24.1%)	15 (22.1%)	
50-59	68 (34.9%)	22 (32.4%)	
≥ 60	80 (41.0%)	31 (45.6%)	
Range	20-85	33-83	
Mean ± SD^b^	56.9 ± 12.0	57.7 ± 11.6	0.649
Sex			
Male (92)	67 (34.4%)	25 (36.8%)	0.720
Female (171)	128 (65.6%)	43 (63.2%)	
Comorbidities			
Number^b^	2.3 ± 0.8	3.0 ± 1.0	<0.001
Hypertension	59 (30.3%)	48 (70.6%)	<0.001
Dyslipidemia	90 (46.2%)	65 (95.6%)	<0.001
Cardiac disease	33 (16.9%)	9 (13.2%)	0.475
Cerebrovascular disease	4 (2.1%)	0 (0.0%)	0.234
Renal disease	29 (14.9%)	9 (13.2%)	0.741
Hypothyroidism	28 (14.4%)	8 (11.8%)	0.592
Others	4 (2.1%)	0 (0.0%)	0.234
Diabetes Duration^b^ (years)	1.21 ± 0.3	1.16 ± 0.3	0.238
Body weight (Kg)^b^	82.9 ± 17.6	74.8 ± 14.3	<0.001
Glycemic control^‡^			
HbA1c (%)	11.2 ± 1.4	10.1 ± 1.6	<0.001
HbA1c (mmol/mol)	99 ± (84–114)	87 ± (69–104)	
Fasting blood glucose (mmol/l)	12.3 ± 4.6	12.2 ± 4.3	0.865
Blood lipids (mmol/l)^‡^			
Total cholesterol	4.7 ± 1.3	5.0 ± 4.1	0.292
Triglycerides	1.9 ± 1.9	1.6 ± 0.7	0.102
HDL cholesterol	1.2 ± 0.3	1.1 ± 0.3	0.506
LDL cholesterol	2.7 ± 1.0	2.7 ± 0.9	0.979
Blood pressure (mmHg)^b^			
Systolic	130.1 ± 13.3	129.1 ± 14.1	0.600
Diastolic	73.2 ± 8.0	74.0 ± 8.2	0.481

^a^Using Chi-square unless mentioned otherwise

^b^Student *t*-test

Diabetes management in the two study groups is described in Additional file 1: Table S1. The patients in the intervention group had significantly higher insulin use (97.4% versus 63.2%, *p* < 0.001), more mixed insulin types (91.3% versus 33.8%, *p* < 0.001), more insulin with multiple daily doses (*p* < 0.001), and more total insulin daily dose per kg (1.3 ± 0.7 versus 0.6 ± 0.4, *p* < 0.001) compared with the control group. They also had significantly higher total number of visits (11.9 ± 6.6 versus 5.1 ± 4.8, *p* < 0.001), as well as visits to a case manager, diabetes educator, and health educator compared with the control group.

The enrollment and final levels, as well as the changes in the study outcomes, are shown in Table 2. In the intervention group, there were significant decreases in the percentage of change relative to baseline in the levels of HbA1c (−27.08%, *p* < 0.001), FBG (−17.0%, *p* < 0.001), total cholesterol (−9.93%, *p* < 0.001), LDL cholesterol (−11.44%, *p* = 0.005), systolic BP (−1.49.0%, *p* = 0.046), and diastolic BP (−3.410%, *p* < 0.001) but significant relative increase in body weight (3.72%, *p* < 0.001). In the control group, there were no significant changes during the study in the levels of different outcomes with the exception of the reduction in total cholesterol (−4.10%, *p* = 0.007). Moreover, the reductions in HbA1c, FBG, total cholesterol, and, to a lesser extent, LDL cholesterol, as well as the increase in body weight, observed in the intervention group were significantly higher than respective changes in the control group.

**Table 2:** Paired post-pre changes (as a percent of the baseline) in HbA1c, blood glucose and lipids, blood pressure and body weight among patients in the intervention and control groups

	Mean ± SD of difference of relative changes related to start of the study		Lower confidence	Upper confidence	Paired t-test	df	*P*-value*
Intervention							
HbA1c	−27.08 ± 12.90		−25.26	−28.90	−29.31	194	<0.001*
FBG	−17.10 ± 43.38		−10.91	−23.29	−5.45	190	<0.001*
Cholesterol	−9.93 ± 19.05		−7.19	−12.66	−7.16	188	<0.001*
Triglycerides	−3.84 ± 50.39		3.35	−11.03	−1.05	190	0.294
HDL	6.56 ± 51.96		13.98	−0.85	1.75	190	0.082
LDL	−11.44 ± 56.35		−3.46	−19.42	−2.83	193	0.005*
Systolic BP	−1.49 ± 10.36		−0.03	−2.95	−2.01	194	0.046*
Diastolic BP	−3.41 ± 12.34		−1.67	−5.15	−3.86	194	<0.001*
Body weight	3.72 ± 5.62		4.54	2.91	9.01	184	<0.001*
Control							
HbA1c	18.75	122.04	48.29	−10.79	1.27	67	0.210
FBG	−0.74	49.16	11.16	−12.64	−0.12	67	0.901
Cholesterol	−4.10	11.28	−1.16	−7.04	−2.79	58	0.007*
Triglycerides	0.73	37.52	−9.82	8.35	0.16	67	0.873
HDL	−0.21	13.56	3.07	−3.49	−0.13	67	0.897
LDL	−1.65	41.20	8.33	−11.62	−0.33	67	0.743
Systolic BP	1.18	10.48	3.72	−1.35	0.93	67	0.356
Diastolic BP	−0.11	12.19	2.85	−3.06	−0.07	67	0.944
Body weight	0.16	4.44	1.24	−0.92	0.30	66	0.766

(*) Statistically significant at *p* < 0.05. The relative change was calculated as the mean change during the study divided by the mean at enrollment

**Fig. 3:**
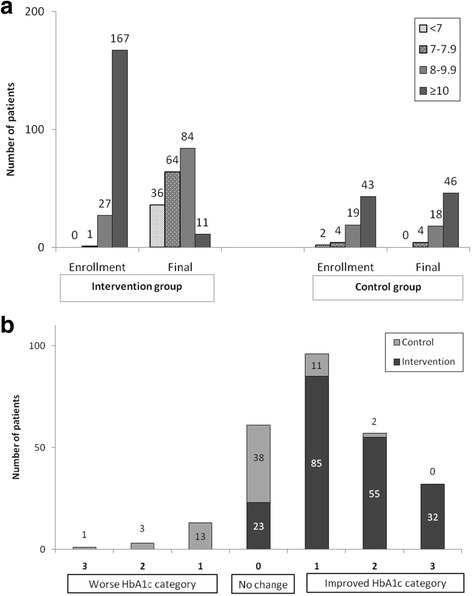
**a** Comparison of different categories of HbA1c at enrollment and final stage of follow up. **b** Change in HbA1c category for both the intervention and control cohorts

The change in HbA1c in both study groups is further illustrated in Fig. 3a and b. In the intervention group, there was a clear reduction in the number of patients with HbA1c ≥10% (86 mmol/mol) (from 167 patients at enrollment to only 11 patients after intervention). This was accompanied by an increase in the number of patients with HbA1c <7% (53 mmol/mol) (from none to 36 patients). The number of the patients with different HbA1c categories in the control group remained relatively constant during the study (Fig. 3a). None of the patients in the intervention group had a worsened HbA1c and those who had a two- or three-category improvement of their HbA1c were all patients in the intervention group, except for two participants. Those who had no change in their HbA1c category represented 12% (*n* = 23) of the intervention group and 56% (*n* = 38) of the control group (Fig. 3b).

The potential correlates of HbA1c improvement were examined in Table 3. In both groups combined, such improvement was positively and significantly correlated with the number of insulin types used, total insulin daily dose per kg, number of all medications used, and total number of clinic visits (including visits to the case manager, diabetes educator, and health educator) but negatively and significantly correlated with the number of comorbidities and visits to a doctor or dietitian. When each group was examined separately, improvement was positively and significantly correlated only with the total number of clinic visits and visits to a case manager in the intervention group but none in the control group. In a multivariate linear regression model that included all significant correlates with HbA1c improvement as shown above, only being in the intervention group (additional 2.47% HbA1c reduction as compared with the control group) and the total number of clinic visits (0.07% HbA1c reduction per visit) were significantly associated with HbA1c improvement (Table 4). These two variables alone explained 35% of the change in HbA1c levels. (Table 5).

**Table 3:** Absolute difference at the end of the study between intervention and control group, showed statistically significant difference in HbA1c, FBG, Cholesterol, LDL and Body weight parameters  with *P* value (<0.001, 0.012, 0.024, 0.033 and <0.001) respectively

Modifiable risk factors	Intervention final measurement at the end of study (mean ± SD)	Control final measurement at the end of study (mean ± SD)	Absolute difference at the end of study between intervention and control	P^†^ Value
HbA1c	8.0 ± 1.2	11.7 ± 11.3	3.7	0.001*
FBG	9.3 ± 4.5	11.2 ± 4.5	1.9	0.005*
Cholesterol	4.1 ± 0.8	4.8 ± 4.1	7	0.022*
Triglycerides	1.6 ± 0.9	1.5 ± 0.6	.1	0.233
HDL	1.2 ± 0.3	1.1 ± 0.3	.1	0.914
LDL	2.1 ± 0.6	2.5 ± 0.9	.4	0.059
Systolic BP	127.5 ± 13	129.7 ± 11.4	2.2	0.223
Diastolic BP	70.3 ± 9.2	73.3 ± 7.8	3	0.215
Body weight	85.7 ± 17.8	74.9 ± 14.8	10.8	0.001*

† − *P* values for the intervention-control differences were estimated using † the Mann–Whitney U test

*P < 0.05

**Table 4:** Correlation between the pre-post changes in HbA1c levels and certain patients’ personal and disease and service characteristics

			Spearman rank correlation			
	Total		Intervention		Control	
	HbA1c (pre-post diff)	HbA1c (pre-post diff % of pre)	HbA1c (pre-post diff)	HbA1c (pre-post diff % of pre)	HbA1c (pre-post diff)	HbA1c (pre-post diff % of pre)
HbA1c (pre-post diff % of pre)	.992§		.984 ^§^		.997^§^	
Age	−0.03	−0.02	−0.03	−0.01	0.07	0.07
Duration of DM	−0.11	−0.10	−0.11	−0.10	−0.54	−0.54
Insulin total dose/kg Number of:	.177^§^	.172^§^	0.00	−0.01	−0.01	−0.02
Comorbidities	−.229^§^	−.232^§^	0.03	0.03	−0.02	−0.01
Insulin types	.423^§^	.427^§^	−0.04	−0.05	0.21	0.21
Oral drugs	0.08	0.07	0.02	0.00	−0.05	−0.03
All medications Number of visits to:	.409^§^	.408^§^	−0.02	−0.04	0.17	0.18
Doctor	−.184^§^	−.180^§^	−0.05	−0.05	0.11	0.11
Case manager	.597^§^	.612^§^	.314^§^	.331^§^	−0.06	−0.07
Dietitian	−.214^§^	−.215^§^	−0.08	−0.08	0.02	0.02
DM educator	.172^§^	.183^§^	0.06	0.08	0.19	0.20
Health educator	.479^§^	.491^§^	0.10	0.10		
Total visits	.552^§^	.569^§^	.303^§^	.321^§^	0.11	0.11

*DM* diabetes mellitus

(§) Statistically significant at *p* < 0.01

**Table 5:** Best fitting multiple linear regression model for the improvement in the level of HbA1c after the intervention

	Unstandardized Coefficients		Standardized Coefficients	t-test	*p*-value	95% Confidence Interval for B	
	B	Std. Error				Lower	Upper
Constant	4.82	0.53		9.09	<0.001	3.77	5.86
Intervention vs control	2.47	0.33	0.45	7.42	<0.001	3.12	1.81
Total number of clinic visits	0.07	0.02	0.22	3.66	<0.001	0.03	0.11

R-square = 0.35, Model ANOVA: F = 60.82, *p* < 0.001. Variables entered and excluded: age, sex, numbers of comorbidities, diabetes duration, insulin and oral medications, and insulin dose

## Discussion

A successful integrated diabetic care program was achieved among a group of patients with poorly controlled T2DM. The program resulted in a considerable improvement of glycemic control and, to a lesser extent, cardiovascular risk factors. Similarly, a number of studies reported improved glycemic control and cardiovascular risk profile at primary care settings after implementing a multidisciplinary care approach, intensifying patient education and modifying workflow to allow better access [15, 19, 20].

The observed reduction in HbA1c in the current study (3.1% absolute and 27.08% relative) was higher than observed in similar studies in the primary care setting [15, 19, 20]. Additionally, a review of studies that implemented intensified diabetic care by a multidisciplinary team, including a primary care physician and clinical pharmacist with an advanced practice nurse, showed between 0.4 and 2.1% improvement in HbA1c levels [12].

The observed higher improvement in the current study may be explained by the relatively worse diabetic control at enrollment (approximately 85% of the intervention group had HbA1c ≥10 [86 mmol/mol]). Those with poor diabetic control in the current study and other studies were the highest group to benefit from the integrated care program [21]. Nevertheless, comparisons of the current findings with other studies may be methodologically challenging as the intensity and frequency of the care provided as well as the composition of the multidisciplinary team markedly varied between different studies. We believe that the observed reduction in HbA1c, if maintained, can result in considerable reductions in cardiovascular morbidity and mortality, as well as the cost of diabetic care [22, 23].

The lack of glycemic control and poor control of cardiovascular risk factors among patients in the control group was not surprising. The majority of Saudi patients with T2DM who receive regular diabetic care at primary care centers, outpatient clinics of internal medicine, or specialized diabetes centers were shown to have poor diabetes control, with the ADA standards of diabetic care not met [24–26]. Several challenges to proper diabetes management in primary care setting have been described. These include insufficient patient education, inadequate patient adherence to medication, infrequent clinic visits, lack of social support, lack of home blood glucose monitoring, inadequate physician attitude and approach, and system barriers [27–29]. The integrated care program described in this study was designed to deal with all the above challenges.

The more frequent clinic visits were the only strategy to predict improved HbA1c levels independently in the current study. It appeared that several other components of the integrated care program that were correlated in univariate analysis to improved HbA1c levels, such as appropriate insulin types and doses, are only working through multiple clinic visits. Additionally, the contribution of clinical pharmacist, who worked as the case manager, may have improved insulin intensification that is not usually tackled by primary care physician [30, 31].

The integrated care program in the current study was associated with a considerably better lipid profile and a slight reduction in BP. Similar findings have been reported before with a considerable increase in the number of those with controlled total and LDL cholesterol and those with controlled BP after a multidisciplinary care [15, 19, 32–34]. However, the percentage changes in BP and, to a lesser extent, blood lipids in the current study were less remarkable compared with glycemic control, probably indicating the need for more involvement by dietitians, especially given that visits to dietitians were less frequent compared with other team members and were not different between the intervention and control groups. It should also be mentioned that the modest increase in body weight that was observed in the intervention group in this study and other studies may be related to increased insulin use among these patients [20].

### Limitations and strengths

The current study has the advantages of examining the effects of a multidisciplinary, multifaceted integrated care program on multiple outcomes and detecting the predictors of improved glycemic control, using an appropriate sample size and controlled design. Findings showed the impact of diabetes care conducted at the primary care level was an appropriate model of care. Nevertheless, we acknowledge some limitations, such differences between the two groups at enrollment. However, these differences were not in one direction, were less clinically meaningful, and probably had no effect on the study findings. For example, the patients in the control group, who had slightly more comorbidities, had slightly better glycemic control. Moreover, the differences in hypertension and dyslipidemia were not associated with differences in BP or blood lipids. The lack of blindness for both patients and care providers may contribute to bias in the results. We tried to minimize such effects by blinding the results to the outcomes assessors (i.e., labs workers and nurses). Additionally, further research encouraged to conduct to evaluate health economic during implementation of integrated care program through multidisciplinary team approach.

## Conclusions

In conclusion, the implementation of a patient-tailored, integrated care program involving a multidisciplinary team approach, frequent clinic visits, and intensified insulin treatment in a primary care setting was associated with marked improvement in glycemic control, modest improvements in blood lipids, and a slight non-significant improvement in BP. Those with poor glycemic control are the highest group to benefit from such integrated care program.

## Additional file

Additional file 1: Table S1. Diabetes treatment in the intervention and control groups. (DOCX 14 kb)

## Abbreviations

BP: Blood pressure
FBG: Fasting Blood Glucose
T2DM: Type 2 Diabetes Mellitus
WHC: Wazarat Healthcare Center
