# Supplementary material for: Impact of an integrated care program on glycemic control and cardiovascular risk factors in patients with type 2 diabetes in Saudi Arabia: an interventional parallel-group controlled study
Source: BMC Fam Pract. 2018 Jan 2;19:1. doi: 10.1186/s12875-017-0677-2 (PMC5748946; doi:10.1186/s12875-017-0677-2)
Supplement: Additional file 1: Table S1. — Diabetes treatment in the intervention and control groups. (DOCX 14 kb) [file 12875_2017_677_MOESM1_ESM.docx]

**Table S1: Diabetes treatment in the intervention and control groups**

|  | **Intervention**  **(N=195)** | **Control**  **(N=68)** | **p-value**^1^ |
| --- | --- | --- | --- |
| **Insulin administration** |  |  |  |
| No | 5 (2.6%) | 25 (36.8%) | <0.001 |
| Yes | 190 (97.4%) | 43 (63.2%) |  |
| Number of insulin types | 1.7±0.5 | 0.7±0.5 | <0.001 |
| **Insulin types** |  |  |  |
| **Unmixed insulin** | **12 (6.2%)** | **20 (29.4%)** | <0.001 |
| Aspart | 1 (0.5%) | 0 (0.0%) |  |
| Regular | 0 (0.0%) | 2 (2.9%) |  |
| Iso | 0 (0.0%) | 6 (8.8%) |  |
| Lantus | 11 (5.6%) | 12 (17.6%) |  |
| **Mixed insulin** | **178 (91.3%)** | **23 (33.8%)** |  |
| Lantus / Aspart | 103 (52.8%) | 1 (1.5%) |  |
| Lantus / Regular | 3 (1.5%) | 0 (0.0%) |  |
| NPH / Aspart | 2 (1.0%) | 0 (0.0%) |  |
| NPH / Regular | 2 (1.0%) | 0 (0.0%) |  |
| Mixtard | 34 (17.4%) | 22 (32.4%) |  |
| Mixtard / Aspart | 22 (11.3%) | 0 (0.0%) |  |
| Mixtard / Regular | 12 (6.2%) | 0 (0.0%) |  |
| Total insulin daily dose/kg | 1.3±0.7 | 0.6±0.4 | <0.001 |
| Number of oral hypoglycemic drugs | 1.0±0.7 | 0.8±0.4 | 0.138 |
| Number of all used medications | 2.7±0.7 | 1.5±0.5 | <0.001 |
| Total number of clinic visits | 11.9±6.6 | 5.1±4.8 | <0.001 |

^*^ Using Chi-square for categorical and Mann–Whitney U test for continuous variables
